# Advanced Ethylene-Propylene-Diene (EPDM) Rubber Composites Filled with Raw Silicon Carbide or Hybrid Systems with Different Conventional Fillers

**DOI:** 10.3390/polym14071383

**Published:** 2022-03-29

**Authors:** Dominik Bartosik, Bolesław Szadkowski, Małgorzata Kuśmierek, Przemysław Rybiński, Ulugbek Mirkhodzhaev, Anna Marzec

**Affiliations:** 1 Institute of Polymer and Dye Technology, Faculty of Chemistry, Lodz University of Technology, Stefanowskiego 16, 90-537 Lodz, Poland; 224011@edu.p.lodz.pl (D.B.); malgorzata.kusmierek@dokt.p.lodz.pl (M.K.); 2 Institute of Chemistry, The Jan Kochanowski University, Uniwersytecka 7, 25-369 Kielce, Poland; przemyslaw.rybinski@ujk.edu.pl; 3 Department of Biophysics, National University of Uzbekistan, Tashkent 100174, Uzbekistan; u.z.mirkhodjaev@gmail.com

**Keywords:** elastomer composites, ethylene-propylene-diene rubber, silicon carbide, mineral fillers, polymer composite properties, hybrid fillers

## Abstract

We studied the effects of silicon carbide (SiC) and SiC hybrid systems with different conventional fillers (silica, carbon black, graphene, hydrotalcite, halloysite) on the rheometric measurements, crosslink density, mechanical performance, aging stability, morphology, thermal behaviour, and flammability of ethylene-propylene-diene (EPDM) rubber composites. The hybrid filler systems showed technically promising synergetic effects on the performance of the EPDM composites. A pronounced reinforcing effect in EPDM composites filled with hybrid SiC filler systems was noted. Tensile strength increased in the systems with carbon black, silica, and graphene nanoplatelets, by 21%, 37%, and 68%, respectively, compared to the neat EPDM. Dynamic-mechanical analysis (DMA) revealed a shift of the glass transition temperature (Tg) of EPDM composites towards higher values following the incorporation of hybrid SiC fillers, indicating that the mobility of the macromolecule chains was restricted by the presence of filler particles. Importantly, the application of SiC as a filler in EPDM rubber composites contributed to a considerable reduction in flammability, as demonstrated by microscale combustion calorimetry (MCC). The most promising results were obtained for HAL/SiC and LDH/SiC hybrid systems, which produced final composites with high flame retardancy and good mechanical performance. The study highlights the significant potential of SiC and SiC hybrid systems as effective fillers improving the properties of elastomer composites.

## 1. Introduction

Flame retardants are widely used in plastics, coatings, and rubbers to reduce the risks of fire in applications in areas where safety is essential, such as aircraft, building/construction, public transport, and housings for electronic elements. Flame retardants must be incombustible, or at least difficult to ignite and burn [1,2,3]. The main problem with many flame retardant additives used in coatings as well as in plastic and rubber composites is that they release toxic gases during thermal decomposition. This concerns compounds with halogens, phosphorous, and metals. Legislation in industrialized countries has tended towards prohibiting the use of some flame retardants. At the same time, there is a requirement for finished products with limited flammability [4,5]. As a consequence, there is much interest from industry in finding efficient, cost-effective, and environmentally friendly flame retardant systems [6]. Previous reports have focused on the use of hydrated fillers, such as ATH and Mg(OH)_2_, which can act as flame retardants and smoke suppressor in polymer materials [7]. Various attempts have been made in order to improve the flame retardancy of polymers by using different halogen-free compounds as flame retardants like fumaric acid or melamine [8,9,10].

In recent years, inorganic and synthetic nanomaterials, such as carbon nanotubes (CNTs) [11,12], carbon nanofibers (CNFs) [13], graphene oxide (GO) and their derivatives [14,15] layered double hydroxides (LDHs) [16], montmorillonite (MMT) [17,18], and halloysite nanotubes (HNTs) [19,20,21], have also been investigated as environmentally friendly flame retardant alternatives to conventional flame retardant materials. These nanofillers possess excellent char-forming ability as well as the ability to decrease fire risks.

Because of its excellent high tension, high tensile strength, toughness, and resistance to aging properties, EPDM (ethylene-propylene-diene rubber) is widely used in many fields [22,23]. However, like many other thermoplastics, EPDM has caused large-scale pollution and harm to the environment. It is highly flammable and releases large amounts of high-temperature toxic smoke during combustion [24]. Therefore, there is an urgent need for effective flame retardants that can be used in EPDM [25,26,27]. The application of traditional halogen free flame retardants like aluminum trihydrate (ATH) or magnesium hydroxide requires a very high loading of the filler within the polymer materials. High loading of additives (sometimes more than 60 wt. %) is very often necessary to achieve a suitable flame retardancy of final products [28]. The disadvantages of such loading can be avoided by application of hybrid systems containing different fillers, which enhance flame resistance of polymer at the same time providing flexibility and good mechanical of final materials.

To further enhance the flammability and mechanical properties of EPDM, a filler such as SiC could be added to the mixture. Silicon carbide powder is characterized by very favorable properties, such as high thermal conductivity, high thermal stability, high strength and hardness, and good resistance to oxidation [29,30,31]. For these reasons, SiC is used in various applications, both as a separate material and as a filler for high-performance composites. Silicon carbide is widely used in composite materials and semiconducting devices that are subjected to high temperature, high frequency, and high-power conditions. It is also used as a reinforcement in polymer matrix composites based on epoxy resins, polyurethane, and elastomers [32,33,34]. Kueseng and Jacob [35] showed that incorporating SiC nanoparticles into natural rubber (NR) contributed to SiC/NR samples with superior mechanical properties than single-walled carbon nanotubes in natural rubber. However, more work is needed to better understand mechanism of interfacial adhesion between SiC and natural rubber. Anancharoenwong et al. [31] added SiC to NR/EPDM blends to improve the thermal properties of the composites. The tensile strength of the vulcanizates increased with loads of SiC up to 10 phr, after which the further addition of SiC deteriorated the tensile properties of the NR materials. Kumar et al. [30] applied a hybrid filler system containing SiC and carbon fiber (CF) to an ethylene–propylene–diene monomer composite cured by peroxides. After the incorporation of SiC, up to 20 phr and CF up to 10 phr, the composites demonstrated higher tensile properties and elongation at break. Although many studies have examined the properties of EPDM composites, there remains a lack of knowledge and understanding of the effects of different hybrid SiC filler systems on EPDM rubber combustion properties.

Here, we present the novel use of raw SiC and SiC hybrid fillers with carbon black, silica, halloysite, layered double hydroxide, inorganic pigment, or graphene as flame retardants in EPDM composites. It was expected that the use of a combination of different fillers would be a good way to obtain a balance between the properties and cost of the rubber materials. We investigated the impact of the SiC hybrid systems on the rheological, mechanical, and thermal properties of the elastomer materials, as well as on their aging behavior. In particular, we investigated the influence of SiC and SiC hybrid systems on the combustion properties of the composites.

## 2. Materials and Methods

### 2.1. Materials

The list of components and their sources were as follows: ethylene-propylene-diene rubber (EPDM) Keltan 4450S, containing 4.3% ethylidene-norbornene (ENB) and 52% ethylene, from Brenntag Polska (Kedzierzyn-Kozle, Poland); sulfur Siarkopol (Tarnobrzeg, Poland); N-cyclohexyl-2-benzothiazolesulfenamide-CBS Alfa Aesar (Lancashire, United Kingdom); stearin POCH S.A. (Gliwice, Poland); microsized zinc oxide ZnO Huta Bedzin (Bedzin, Poland); silicon carbide SiC Sigma Aldrich, particle size 200–450 mesh; silica Aerosil 380 Evonik Degussa GmbH (Essen, Germany), surface area 350 m^2^/g; Fast Extrusion Furnace Black (FEF, N550)-Carbon black Evonik Degussa GmbH (Essen, Germany), particle size of 39–55 nm; graphene nanoplatelets, XGnP XG Science Inc. (Lansing, MI, USA), surface area 500 m^2^/g, particle size, average particle thickness around 2 nm, average particle diameter >2 μm; hydrotalcite LDH, synthetic Sigma Aldrich, particle size 1 μm; halloysite nanoclay Sigma Aldrich, particle diameter 30–70 nm, length 1–3 μm; Pigment Yellow 119 Kremer Pigmente (Aichstetten, Germany), chemical formula Fe_2_O_3_·ZnO. Eleven rubber composites were prepared, based on the formulations presented in Table 1. Henceforth, the following numbers and abbreviations for the compounds will be used: 1-EPDM; 2-EPDM/5SiC; 3-EPDM/10SiC; 4-EPDM/15SiC; 5-EPDM/20SiC; 6-EPDM/10SiC/5SiO_2_; 7-EPDM/10SiC/5CB; 8-EPDM/10SiC/5XGnP; 9-EPDM/10SiC/5LDH; 10-EPDM/10SiC/5HAL; 11-EPDM/10SiC/5PY119.

### 2.2. Methods

The ethylene-propylene-diene rubber composites were blended in an open laboratory two-roll mill. The length of the rolling mill was 450 mm and the diameter of 200 mm. The compounding temperature was kept at around 40 °C and each composite was mixed for about 20 min. The curing parameters of the elastomer compounds were determined using a MonTech D-RPA 3000 Moving Die Rheometer (MonTech, Buchen, Germany). For each composite the following were measured: scorch time-t_02_; optimum cure time-t_90_; maximum elastic torque-M_max_; minimum elastic torque-M_min_; increment of elastic torque ΔM = M_max_ − M_min_. Based on the results, the blends were vulcanized on a hydraulic press for the curing time (t_90_) determined in rheometric measurements. The equilibrium swelling method was employed to evaluate the crosslink density of the EPDM vulcanizates, according to the ISO 1817 standard procedure. Four differently shaped samples, weighing in the range of 20–30 mg, were used for swelling experiments in toluene solvent. The crosslink density was calculated based on the Flory-Rehner equation [36,37]:

(1) $$\upsilon_{e}=\frac{\ln\left(1-V_{r}\right)+V_{r}+{\mu V}_{r}}{V_{s}d_{r}\left(\frac{V_{2}}{2}-V_{2}^{\frac{1}{3}}\right)}$$

where $\upsilon_{e}$ is the chemical cross-link density, V_s_ is the molar volume of the solvent, V_r_ is the volume fraction of the rubber in the swollen specimen, d_r_ is the density of the rubber, and  μ is the interaction parameter. The mechanical properties of each composite were examined using a universal tensile testing machine Zwick/Roell 1435 (Zwick Roell Group, Ulm, Germany). The elongation at break and the tensile strength were calculated as the arithmetic averages of five measurements. The same procedures were performed on thermo-oxidative aged samples. The vulcanized composites were kept for three weeks in a dryer set to 60 °C. Each week, a piece of the vulcanizate was cut off, large enough to perform at least three measurements on a Zwick/Roell 1435 tensile testing machine. The aging coefficient was determined based on changes in the mechanical parameter, using the following Equation (1) [38]:

(2) $$\mathrm{Aging}\;\mathrm{coefficient}=\frac{{\left(T_{S}\cdot E_{B}\right)}_{\mathrm{after}\;\mathrm{aging}}}{{\left(T_{S}\cdot E_{B}\right)}_{\mathrm{before}\;\mathrm{aging}}}$$

where $T_{S}$ means the tensile strength (MPa) and $E_{B}$(%) is an elongation at break of the vulcanizate. The morphology of the samples was examined and photographed using a scanning electron microscope (SEM, Zeiss, ULTRA Plus, Oberchoken, Germany). The flammability of the EPDM composites was evaluated using a microscale combustion calorimetry test (MCC). The MCC test was performed on 2.5 mg samples on a micro-calorimeter (Fire Testing Technology Limited) equipped with a pyrolyzer (at 750 °C) and combustor (at 900 °C).

## 3. Results

### 3.1. Rheometeric and Crosslink Density Measurements

**Figure 1:**
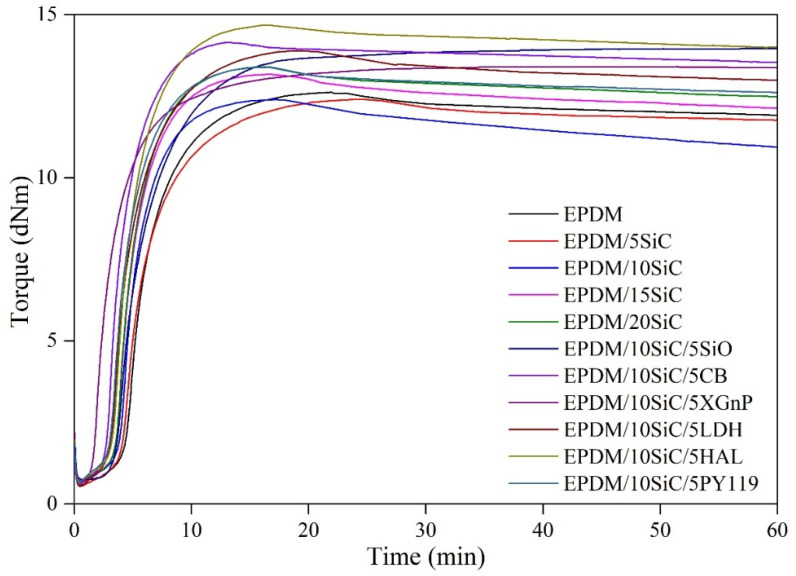
Rheometric curves for EPDM rubber compounds filled with SiC or SiC hybrid systems.

**Figure 2:**
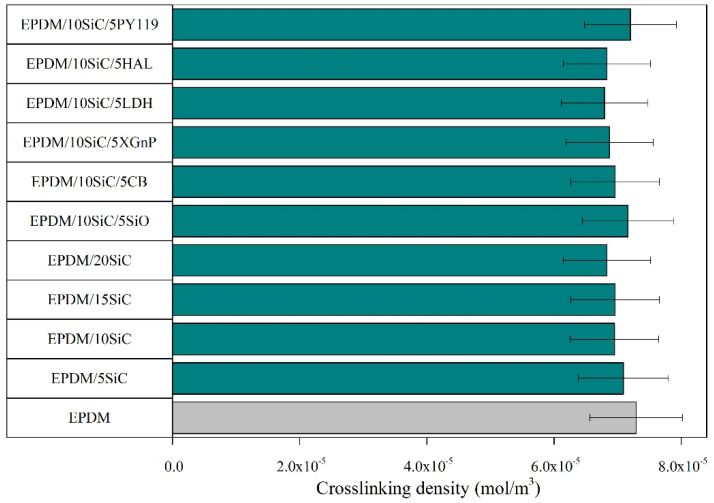
Cross-link density of EPDM vulcanizates filled with SiC or SiC hybrid systems.

The effect of the SiC filler and its hybrid systems used on the curing behavior of the prepared EPDM compounds was investigated in rheometric measurements. The curing curves and corresponding rhemoetric results are presented on Figure 1 and Table 2, while crosslink density values determined for studied vulcanizates are depicted on Figure 2. The rheometric characteristics showed that the incorporation of SiC slightly increased the M_min_ value, which was related to the change in viscosity. This effect is typical for elastomer blends filled with rigid filler particles [39,40]. The highest M_min_ values were observed for uncured EPDM/10SiC/5SiO_2_ sample containing silica (0.76 dNm) and for EPDM/10SiC/5XGnP filled with graphene (0.72 dNm). This could be related to the particle size of the material. Furthermore, it can be seen that incorporation of the SiC filler into EPDM mixes did not contribute to the changes in the increment of torque (ΔM). This can most likely be explained by the relatively low specific surface area of the microsized SiC filler and its poor dispersion in the elastomer matrix, leading to low adhesion with EPDM. On the other hand, the presence of active fillers in the SiC hybrid filler systems produced EPDM composites with higher ΔM parameters. For instance, the ΔM parameter values for EPDM/10SiC/5SiO_2_, EPDM/10SiC/5CB, and EPDM/10SiC/5HAL were about 10%, 12%, and 16% higher, respectively, than the value for unfilled EPDM. This is a common effect observed in rubber compounds filled with reinforcing fillers [41,42]. It should also be noted that all the filled EPDM blends exhibited reduced scorch time (t_05_). Increasing the concentration of SiC in the EPDM compounds caused a progressive reduction in the t_05_. The samples with graphene EPDM/10SiC/5XGnP and the sample with carbon black EPDM/10SiC/5LDH showed the shortest t_05_ of the composites with mixed additives. The scorch time of the uncured compound with graphene EPDM/10SiC/5XGnP fell from 4.66 min (for the reference EPDM sample) to 1.91 min. These decreases were caused by the particular properties of the additives: both graphene and carbon black exhibit thermal conductivity, which results in enhanced heat transfer inside the tested sample, leading to a decrease in t_05_ [43,44].

One of the crucial parameters determining the profitability of the vulcanization process, especially on an industrial scale, is the vulcanization time t_90_. Based on our previous study [45], in which EPDM was used as the polymer matrix, we decided to raise the vulcanization temperature to 180 °C. As expected, raising the vulcanization temperature shortened the process from 28.75 min for the reference to 11.01 min. Each of the additives effectively decreased vulcanization time, except for EPDM/10SiC/5SiO_2_. This may have been caused by the formation of larger agglomerates inside the vulcanizate. The sulfur crosslinking system could also partially be adsorbed onto the outer surface of the silica, which resulted in a slower curing process. Moreover, silica is characterized by low thermal conductivity, which may lead to deterioration of heat transfer in the sample and prolongation of curing time. On the other hand, the shortest vulcanization time was observed for the compounds containing SiC and CB fillers, which has favorable thermal conductivity.

EPDM rubber can be effectively cured using both the conventional crosslinking system and peroxides due to the presence of unsaturated bonds in its structure. In our study, sulfur was used for crosslinking, as well as CBS, which is a sulfenamide characterized as an accelerator with relatively fast curing speed. The similar crosslinking density for EPDM cured by peroxides to the reference sample EPDM in our study has been previously reported in other work [46]. The comparable values proved the effectiveness of the selected crosslinking system. We observed that the crosslinking density values was visibly reduced with increasing concentrations of SiC. The lower crosslinking density of the vulcanizates containing layered minerals (such as halloysite EPDM/10SiC/5HAL or hydrotalcite EPDM/10SiC/5LDH) and carbon fillers probably resulted from partial adsorption of the curatives on the surface of the additives, which decreased the effectiveness of crosslinking process.

### 3.2. Mechanical Properties and Morphology of EPDM Composites

**Figure 3:**
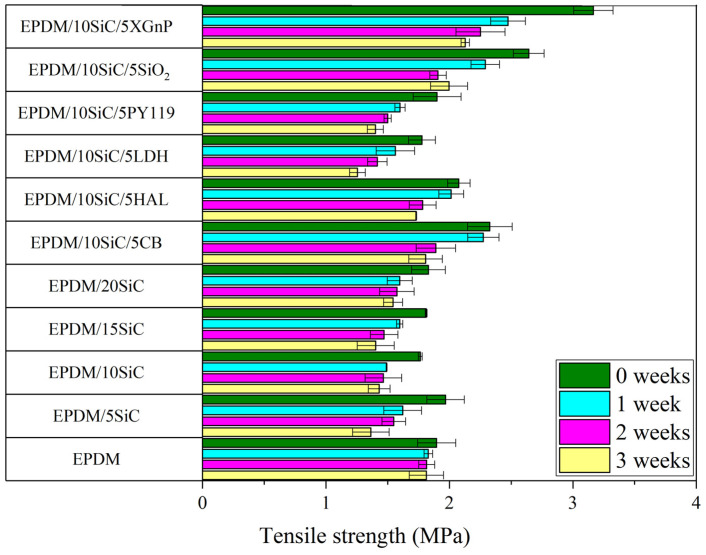
Tensile strength of composites before and after thermo-oxidative ageing.

**Figure 4:**
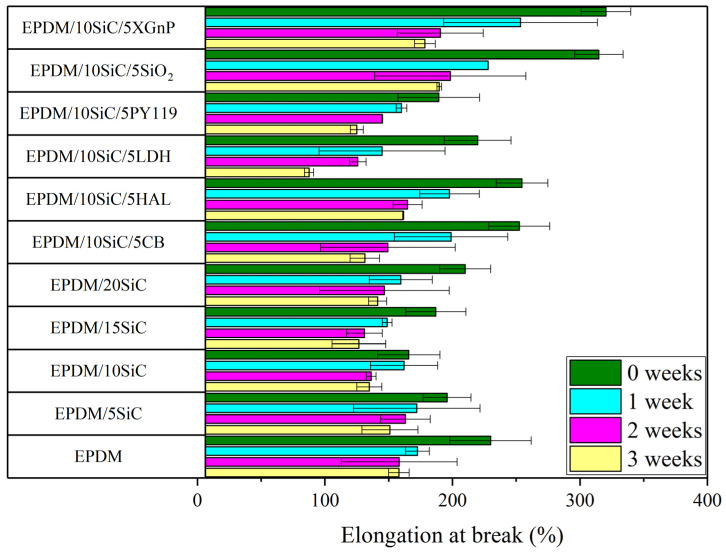
Elongation at break of composites before and after thermo-oxidative ageing.

**Figure 5:**
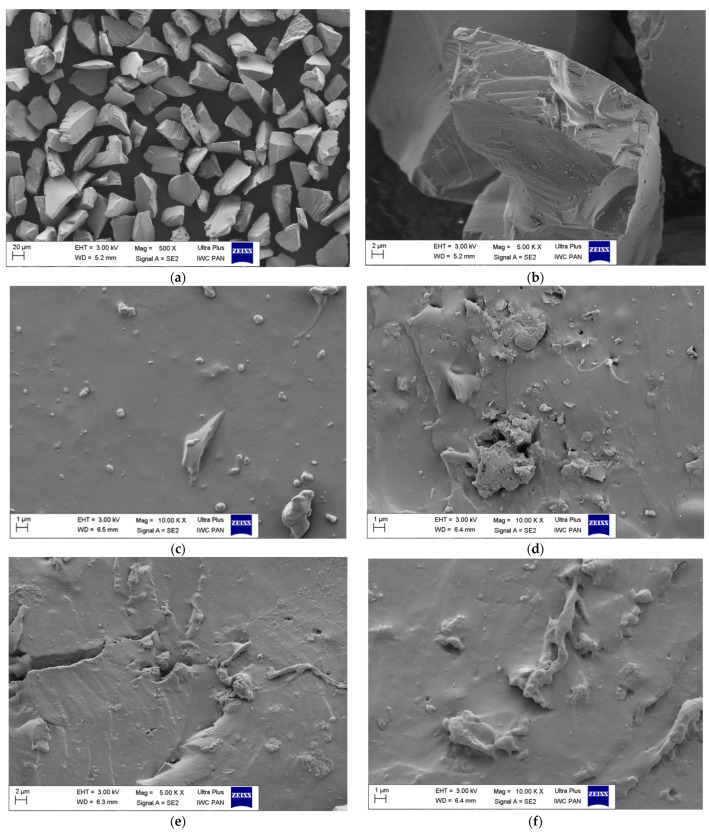

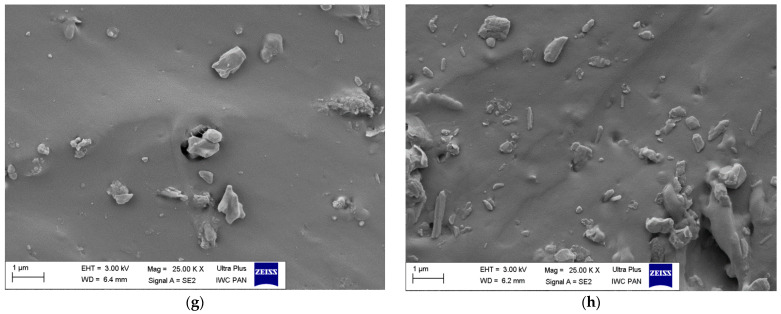
Scanning electron microscopy (SEM) microphotographs of pure SiC filler particles (**a**,**b**) and EPDM vulcanizates containing: (**c**) 5 phr of SiC; (**d**) 15 phr of SiC; (**e**) 10 phr of SiC and 5 phr of SiO_2_; (**f**) 10 phr of SiC and 5 phr of CB; (**g**) 10 phr of SiC and 5 phr of xGnP; (**h**) 10 phr of SiC and 5 phr of HAL.

The static mechanical properties (tensile strength and elongation at break) of the EPDM-filled composites are presented in Figure 3 and Figure 4. As can be seen, raw silicon carbide filler combined with EPDM rubber exhibited worse properties than EPDM on its own. The tensile strength of the EPDM rubber decreased with increasing amounts of SiC. This was due to the agglomeration of the SiC particles in the EPDM matrix, as evidenced by SEM analysis (Figure 5c,d). The agglomerates formed by SiC particles acted as points of high stress, which were prone to breakage. This caused local defects and contributed to mechanical failure. Filler dispersion and interfacial interactions are key factors that determine the final properties of rubber composites. Lack of enhancement of the mechanical properties of the EPDM/SiC samples may indicate poor interactions and low adhesion between the filler particles and the rubber matrix [47]. The reduction in crosslink density following the incorporation of SiC may also have contributed slightly to the lower tensile strength of the EPDM composites. Similar observations were made by Shiva et al. [48], who reported that application of 18 phr of silicon carbide in butyl rubber/carbon black composites led to decrease in tensile strength from 11.3 MPa to 9.9 MPa.

**Figure A1:**
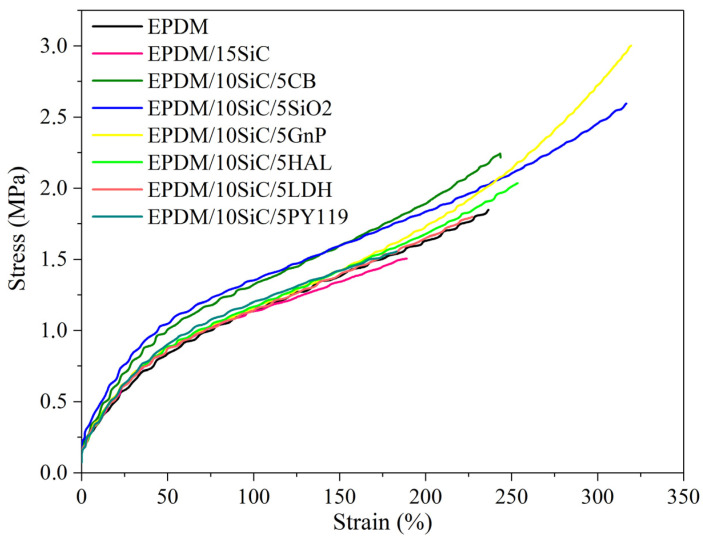
Stress-strain curves of the EPDM composites filled with different hybrid SiC filler systems.

Increasing the concentration of SiC in the rubber also caused a reduction in elongation at break (EB), due to the increased viscosity and stiffness of the filled composites. For example, EPDM composite filled with 20 phr of SiC reached an EB of 210%, whereas for neat EPDM EB was 230%. The hybrid SiC filler systems with silica, carbon black, and graphene significantly improved the mechanical properties of the EPDM composites. All these systems strengthened the EPDM rubber, as shown by the increases in tensile strength (by 33% for SiO_2_, 17% for CB, 59% for xGnP compared to neat EPDM). This can be clearly seen on stress-strain curves of the studied composites presented in Figure A1 (Appendix A). These increases were due to the high reinforcing activity of the added fillers in the hybrid systems. Kumar et al. [49] investigated graphite powder and precipitated silica as binary fillers in natural rubber composites. Filler dispersion was a critical parameter. The authors observed that, as a solid lubricant, graphite powder promoted the dispersion of the silica filler in the rubber matrix. According to the study, graphene and carbon black can also influence the dispersion of SiC in EPDM matrix, contributing to better mechanical properties in samples containing hybrid systems. No mechanical reinforcement was observed in the cases of halloysite, hydrotalcite, or pigment PY119.

The morphology of each elastomer composite and the degree of dispersion of the SiC and SiC hybrid filler systems in the EPDM matrix were studied using SEM. Figure 5a,b show the morphology of the raw SiC filler particles while Figure 5c–h present the distribution of studied filler systems in EPDM matrix. One can see that SiC particles exhibit a brick-like morphology with basal dimensions of about 20–30 µm. The dispersion of the fillers in the EPDM matrix was found to vary considerably. The SiC particles appear to strongly agglomerate in the rubber matrix, which probably explains the reduction in the mechanical strength of the composites. The SEM images of EPDM composites in Figure 5e–h show rather smooth surfaces with some areas of filler agglomeration. All the EPDM-filled composites show some small agglomerations of fillers. The largest clusters, with average lengths of about few microns, can be seen in the case of the EPDM composite containing 20 phr of SiC indicating poor dispersion of that filler (Figure 5d). On the other hand, the EPDM composites filled with hybrid filler systems show more uniform dispersion with smaller agglomerates formed by filler particles in polymer matrix. Based on these results, the application of binary SiC systems with other mineral fillers may enhance its dispersion in elastomer matrix, however, further studies are still needed on improving the compatibility between SiC and rubber matrix.

**Figure 6:**
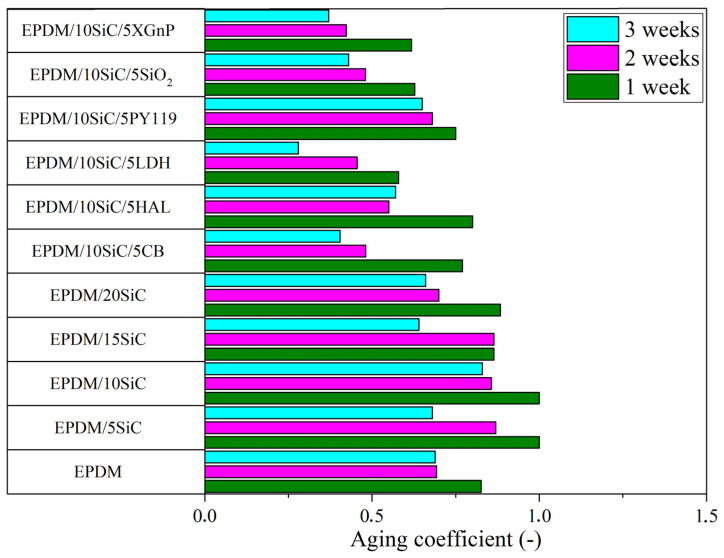
Aging coefficient calculated for composites before and after thermo-oxidative ageing.

In the next part of this study, we investigated the influence of SiC and SiC hybrid filler systems on the resistance of EPDM vulcanizates to thermo-oxidative aging. The EPDM composites were stored at 70 °C for up to 3 weeks. Their tensile properties were tested and compared with the non-aged vulcanizates. The tensile strength, elongation at break, and aging coefficients of the studied samples are presented in Figure 3, Figure 4 and Figure 6. As expected, prolonged exposure to thermo-oxidation caused a progressive reduction in the tensile strength and elongation of the vulcanizates. This was most likely the effect of the increased crosslinking density, which led to worse mechanical properties after exposure to high temperatures. As can be seen in Figure 6, the reference EPDM composites showed aging factors of approximately 0.80, 0.62, and 0.60 after 1, 2, and 3 weeks, respectively. Therefore, they were relatively susceptible to thermo-oxidative aging. The SiC hybrid systems with XGnP, SiO_2_, PY119, LDH, HAL, and CB slightly reduced the resistance of EPDM to thermo-oxidative aging, as evidenced by lower aging factors. On the other hand, the application of raw silicon carbide filler contributed to improve aging resistance, especially when SiC loading was in the range of 5–10 phr. Similar results were reported by Anancharoenwong et al. [31] for NR/EPDM blends filled with different concentrations of silicon carbide.

### 3.3. Dynamic Mechanical Analysis (DMA)

**Figure 7:**
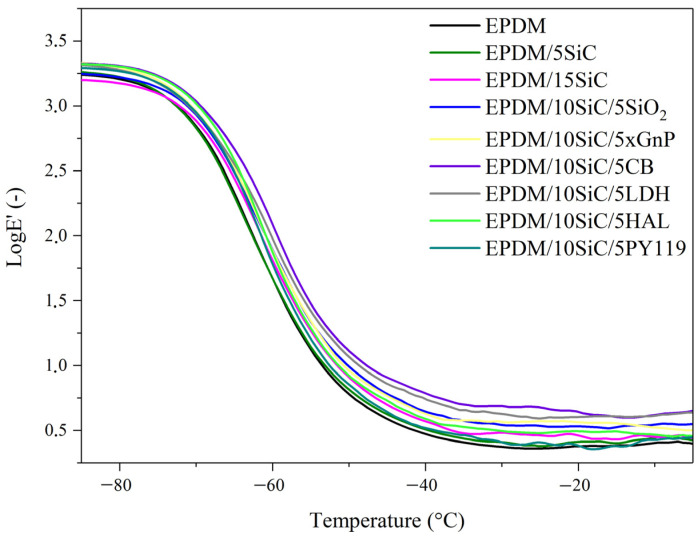
Storage modulus versus temperature curves obtained in DMA measurements for EPDM-filled composites.

**Figure 8:**
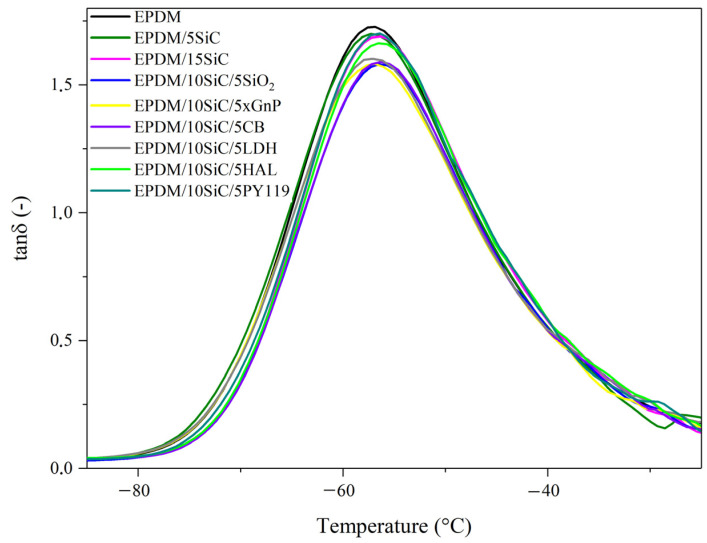
Tan δ versus temperature curves from DMA measurements for EPDM-filled composites.

The dynamic mechanical properties of the EPDM-filled composites were investigated by dynamic-mechanical analysis (DMA). The resulting curves are presented in Figure 7 and Figure 8. The corresponding data are given in Table 3 and Table 4. With increasing SiC content the storage modulus (E’) of the EPDM at three temperatures (−80 °C, 0 °C, and 20 °C) increases slightly. The application of silica, graphene, or halloysite in the hybrid filler systems resulted in a further increase in E’, due to the reinforcing effect of the additives. For example, the storage modulus at 20 °C increased from 2.44 MPa for neat EPDM to 3.68 MPa, 3.92 MPa, and 4.57 MPa for composites filled with XGnP, SiO_2_, and CB, respectively. This reinforcement was further reflected in the decrease in the maximum tan δ values (Figure 8) and in the improved tensile properties (Figure 3) of the studied composites. These results indicate that the vulcanizates had strong rubber-filler interactions and a developed rubber network structure. The slight shift in glass transition temperatures (Figure 8 and Table 3) to higher values reflects the fact that the presence of reinforcing additives may restrict the movement of rubber chain segments.

### 3.4. Flammability and Thermal Stability of EPDM Composites

**Figure 9:**
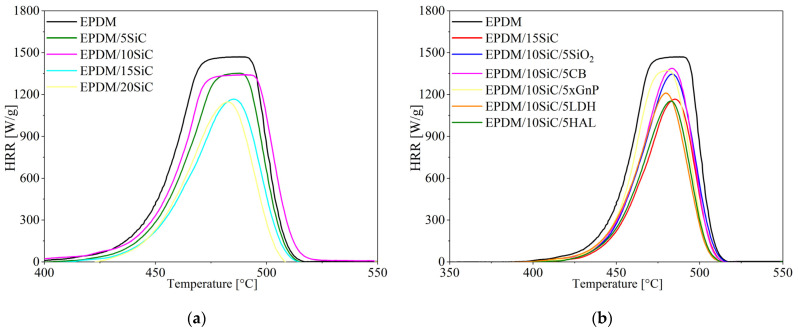
HRR curves of the EPDM composites filled with SiC (**a**) and its hybrid filler systems (**b**).

The flammability of the EPDM-filled composites was investigated using the microscale combustion calorimetry test (MCC), which is known to be suitable for assessing the combustion behavior of elastomer composites. The most important parameter monitored during this test is the heat release rate (HRR). Figure 9 shows changes in HRR as a function of temperature. The other combustion parameters are listed in Table 2. The HRR curves shown in Figure 9 show that increasing the concentration of SiC in the EPDM compounds significantly reduced the peak HRR. The reductions in HRR following the application of SiC were accompanied by a decrease in total heat release (THR) and heat release capacity (HRC), as shown in Table 5. The most pronounced reduction in the combustion parameters was observed for the sample containing 15 phr of SiC. The HRR, THR, and HRC were reduced by about 20%, 41%, and 21%, respectively, compared to the values for neat EPDM. Silicon carbide belongs to a group of fillers that can prevent heat and oxygen transfer at elevated temperatures through a polymer matrix. Therefore, SiC plays the role of a thermal insulator and mass transport barrier in the elastomer matrix, reducing the flammability of the elastomer composite. Bharath Kumar et al. [50] report that 15% of SiC loading in vinylester/glass fiber composites reduces the flammability of polymer composites considerably, due to the increased stability of the char formed during combustion. This promotes the mechanical cohesion of the crust, which reduces the amount of heat transfer to the polymer. Reduced flammability has also been observed for silicon rubber [51] and polyurethane composites [52]. The application of the SiC hybrid filler systems with different mineral fillers reduced the flammability of the EPDM composites. However, the HRR, THR, and HRC parameters were slightly higher for all the hybrid systems compared to EPDM filled with 15 phr of raw silicon carbide. An exception was the composite containing the SiC/HAL filler system, for which the combustion parameters were almost the same as those for the EPDM/15SiC composite. This may have been the result of the highly flame-retardant properties of halloysite, which formed a spatial network impeding the diffusion of thermal decomposition products to flame and oxygen [53].

**Figure 10:**
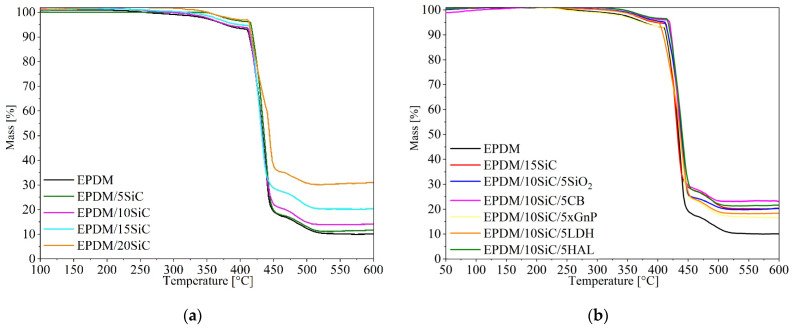
TGA curves for EPDM composites filled with different concentrations of SiC (**a**) and different hybrid filler systems (**b**).

**Figure A2:**
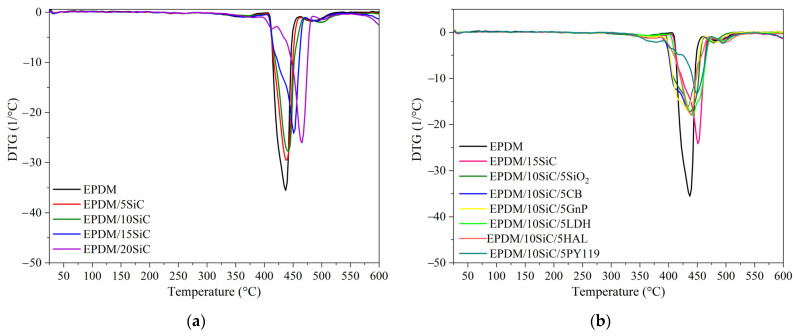
DTG curves of the EPDM composites filled with: (**a**) different concentrations of SiC and (**b**) different hybrid SiC filler systems.

The thermal stability of the EPDM composites filled with different SiC systems was analyzed by thermogravimetric analysis (TGA). The TGA thermograms of the studied composites are presented in Figure 10, while DTG curves are depicted in Figure A2 (Appendix A). Characteristic thermal decomposition temperatures are listed in Table 6. Above 400 °C, the EPDM composites reduced in weight dramatically, due to decomposition. The application of raw SiC as a filler did not cause significant alterations in the thermal stability of EPDM rubber. The hybrid systems of SiC with different mineral fillers also exhibited similar stability at elevated temperatures to unfilled EPDM. However, compared to the pure EPDM elastomer the composites filled with SiC and hybrid filler systems showed slightly higher quasi-steady temperatures, indicating that the EPDM-filled composites were more stable than the reference sample. It is likely that the EPDM matrix was protected from the heat by the SiC particles (similarly to other mineral fillers). Kharazi et al. [54] showed that the presence of nano-SiC particles slightly delays the initiation of thermal degradation and reduces the rate of thermal degradation of butadiene rubber composites. Wang et al. [51] report that the application of hybrid filler system made from silicon carbide and boron nitride can effectively improve the thermal stability of silicon rubber composites. This effect can be explained by the improvement in physical and chemical cross-linking points and the interactions between fillers and the silicone matrix. The application of SiC and its hybrid systems with other mineral fillers in EPDM rubber also contributed to reduce the rate of thermal decomposition (dm/dt) (Table 6). Generally, reducing the decomposition rate of polymeric materials has a positive influence by reducing their flammability. The decrease in the decomposition rate of the studied compounds may result from the barrier properties of the mineral fillers used. The presence of filler particles in the elastomer matrix probably limited the diffusion of small-molecular products outside the matrix during thermal degradation. Increasing the concentration of SiC lead to the formation of increasing amounts of char residue in the studied composites. The increased amount of char residue (P_600_) could also affect the flammability of the composites. Interestingly, the sample containing carbon black showed higher char residue than the sample containing graphene. This was most likely related to the formation of so-called “bound rubber” in the EPDM/10SiC/5CB composite. In contrast, graphene flakes form a layered material comprising sp^2^-bonded carbon atoms in a planar arrangement, separated by an interlayer distance about 0.335. The limited d-spacing in graphite makes it difficult for macromolecules to enter gallery spaces, preventing the formation of bound rubber.

## 4. Conclusions

In this study, a series of EPDM composites filled with SiC and SiC hybrid filler systems were fabricated. The properties of the EPDM/SiC composites were tuned by adjusting the contents of selected conventional fillers used in polymer technology, such as silica, graphene, and carbon black. The use of these reinforcing fillers in the hybrid systems resulted in EPDM composites with improved static and dynamic mechanical properties. The EPDM/SiC composites were also modified by the addition of halloysite nanotubes. The use of SiC in filler systems with halloysite nanotubes resulted in a considerable improvement in the flame retardancy of the EPDM composites. The incorporation of 15 phr of SiC into EPDM caused a reduction in the heat release rate parameter from 1460 W/g for neat EPDM to 1167 W/g for the EPDM/15SiC composite, as measured by MCC tests. The results of this study show that SiC can be successfully used as filler for reducing the flammability of elastomer composites. Elastomer composites filled with SiC hybrid filler systems with different conventional fillers offer an attractive rubber material resistant to fire, thermo-oxidative aging, and mechanical stress. The low concentration of hybrid filler systems in the polymer matrix allows for relatively low production costs, which is often a determining factor in the selection of flame-retardant elastomer products. Future work should focus on improving the dispersion of SiC and its compatibility with the rubber matrix.

## Figures and Tables

**Table 1:** Composite formulations.

Compound	1	2	3	4	5	6	7	8	9	10	11
EPDM	100	100	100	100	100	100	100	100	100	100	100
S	1.5	1.5	1.5	1.5	1.5	1.5	1.5	1.5	1.5	1.5	1.5
CBS	1.5	1.5	1.5	1.5	1.5	1.5	1.5	1.5	1.5	1.5	1.5
Stearin	1	1	1	1	1	1	1	1	1	1	1
ZnO	5	5	5	5	5	5	5	5	5	5	5
SiC	-	5	10	15	20	10	10	10	10	10	10
Silica	-	-	-	-	-	5	-	-	-	-	-
Carbon black	-	-	-	-	-	-	5	-	-	-	-
Graphene	-	-	-	-	-	-	-	5	-	-	-
Hydrotalcite	-	-	-	-	-	-	-	-	5	-	-
Halloysite	-	-	-	-	-	-	-	-	-	5	-
PY119	-	-	-	-	-	-	-	-	-	-	5

phr-parts per hundred rubber.

**Table 2:** Rheometric parameters for EPDM rubber compounds filled with SiC or SiC hybrid systems.

Sample	t_05_ (min)	t_90_ (min)	M_min_ (dNm)	M_max_ (dNm)	ΔM (dNm)
EPDM	4.61	11.01	0.58	12.62	12.04
EPDM/5SiC	4.34	11.81	0.55	12.41	11.87
EPDM/10SiC	4.08	8.06	0.63	12.39	11.69
EPDM/15SiC	3.78	8.08	0.62	13.17	12.55
EPDM/20SiC	3.77	8.53	0.63	13.40	12.77
EPDM/10SiC/5SiO_2_	3.90	11.80	0.76	13.96	13.20
EPDM/10SiC/5CB	2.93	7.42	0.64	14.15	13.51
EPDM/10SiC/5XGnP	1.91	8.53	0.72	13.42	12.70
EPDM/10SiC/5LDH	3.32	9.62	0.54	13.90	13.36
EPDM/10SiC/5HAL	3.45	8.55	0.69	14.67	13.98
EPDM/10SiC/5PY119	3.25	8.19	0.64	13.39	12.75

**Table 3:** Glass transition temperatures (Tg) and maximum tan δ values for different EPDM composites in DMA measurements.

Sample	Tg (°C)	Max. tan δ (−)
EPDM	−56.85	1.73
EPDM/5SiC	−56.55	1.69
EPDM/15SiC	−56.35	1.69
EPDM/10SiC/5SiO_2_	−56.45	1.58
EPDM/10SiC/5XGnP	−56.90	1.58
EPDM/10SiC/5CB	−56.30	1.59
EPDM/10SiC/5LDH	−57.10	1.60
EPDM/10SiC/5HAL	−56.55	1.66
EPDM/10SiC/5PY119	−56.40	1.70

**Table 4:** Storage modulus at different temperatures for EPDM compounds in DMA measurements.

Sample	E’ at −80 °C (MPa)	E’ at 0 °C (MPa)	E’ at 20 °C (MPa)
EPDM	1614	2.21	2.44
EPDM/5SiC	1673	2.54	2.56
EPDM/15SiC	1499	2.71	2.92
EPDM/10SiC/5SiO_2_	1666	3.68	3.92
EPDM/10SiC/5XGnP	1986	3.37	3.68
EPDM/10SiC/5CB	2033	4.37	4.57
EPDM/10SiC/5LDH	1899	3.69	4.08
EPDM/10SiC/5HAL	2007	2.9 8	3.35
EPDM/10SiC/5PY119	1861	2.88	3.01

**Table 5:** Results of microscale combustion calorimetry (MCC) tests on EPDM-filled composites.

Sample	HRR [W/g]	THRR [°C]	THR [kJ/g]	HRC [J/g × K]
EPDM	1460	477	67.9	1421
EPDM/5SiC	1371	488	56.0	1328
EPDM/10SiC	1332	479	65.3	1292
EPDM/15SiC	1167	484	39.7	1126
EPDM/20SiC	1195	482	44.8	1195
EPDM/10SiC/5SiO_2_	1346	483	48.7	1299
EPDM/10SiC/5CB	1387	483	49.6	1341
EPDM/10SiC/5XGnP	1373	480	49.2	1322
EPDM/10SiC/5LDH	1226	480	45.3	1185
EPDM/10SiC/5HAL	1165	482	41.7	1127
EPDM/10SiC/5PY119	1275	481	44.8	1233

**Table 6:** Thermal stability of EPDM-filled composites.

Sample	T_5_ [°C]	T_50_ [°C]	T_R_ [°C]	T_RMAX_ [°C]	dm/dt [%/min]	P_600_ [%]
EPDM	415	435	415	431	31.0	10.1
EPDM/5SiC	375	435	410	431	28.8	11.7
EPDM/10SiC	380	435	410	430	26.8	14.1
EPDM/15SiC	395	430	415	425	29.3	20.3
EPDM/20SiC	415	445	410	440	18.5	30.1
EPDM/10SiC/5SiO_2_	415	440	410	435	25.4	20.2
EPDM/10SiC/5CB	420	440	420	431	31.2	25.1
EPDM/10SiC/5XGnP	375	435	410	435	24.8	16.5
EPDM/10SiC/5LDH	400	435	400	435	19.7	18.3
EPDM/10SiC/5HAL	410	440	415	435	25.5	24.6
EPDM/10SiC/5PY119	405	445	400	435	13.5	22.1

## Data Availability

Data sharing is not applicable for this article.

## References

[1] Irvine D.J., McCluskey J.A., Robinson I.M. (2000). Fire hazards and some common polymers. Polym. Degrad. Stab..

[2] Kausar A., Rafique I., Anwar Z., Muhammad B. (2016). Recent developments in different types of flame retardants and effect on fire retardancy of epoxy composite. Polym. Plast. Technol. Eng..

[3] Braun U., Balabanovich A.I., Schartel B., Knoll U., Artner J., Ciesielski M., Döring M., Perez R., Sandler J.K., Altstädt V. (2006). Influence of the oxidation state of phosphorus on the decomposition and fire behaviour of flame-retarded epoxy resin composites. Polymer.

[4] Lawson D.F. (1986). Recent developments in the flammability of elastomeric materials. Rubber Chem. Technol..

[5] Kuo P.L., Chang J.M., Wang T.L. (1998). Flame-retarding materials—I. Syntheses and flame-retarding property of alkylphosphate-type polyols and corresponding polyurethanes. J. Appl. Polym. Sci..

[6] Cheng X.W., Guan J.P., Tang R.C., Liu K.Q. (2016). Phytic acid as a bio-based phosphorus flame retardant for poly(lactic acid) nonwoven fabric. J. Clean. Prod..

[7] Pinto U.A., Visconte L.L.Y., Gallo J., Nunes R.C.S. (2000). Flame retardancy in thermoplastic polyurethane elastomers (TPU) with mica and aluminum trihydrate (ATH). Polym. Degrad. Stab..

[8] Wang K., Wang J., Zhao D., Zhai W. (2017). Preparation of microcellular poly(lactic acid) composites foams with improved flame retardancy. J. Cell. Plast.

[9] Zhang T., Yan H., Shen L., Fang Z., Zhang X., Wang J., Zhang B. (2014). Chitosan/phytic acid polyelectrolyte complex: A green and renewable intumescent flame retardant system for ethylene-vinyl acetate copolymer. Ind. Eng. Chem. Res..

[10] Tributsch H., Fiechter S., de Wilde W.P., Brebbia C.A. (2008). The material strategy of fire-resistant tree barks. High Performance Structures and Materials IV.

[11] Wang J. (2021). Flame retardancy and dispersion of functionalized carbon nanotubes in thiol-ene nanocomposites. Polymers.

[12] Kashiwagi T., Gruke E., Hilding J., Groth K., Harris R., Awad W., Douglas J. (2002). Thermal degradation and flammability properties of poly(propylene)/carbon nanotube composites. Macromol. Rapid. Commun..

[13] Szadkowski B., Marzec A., Rybiński P. (2020). Silane treatment as an effective way of improving the reinforcing activity of carbon nanofibers in nitrile rubber composites. Materials.

[14] Yuan B., Bao C., Song L., Hong N., Liew K.M., Hu Y. (2014). Preparation of functionalized graphene oxide/polypropylene nanocomposite with significantly improved thermal stability and studies on the crystallization behavior and mechanical properties. Chem. Eng. J..

[15] Huang G., Chen S., Tang S., Gao J. (2012). A novel intumescent flame retardant-functionalized graphene: Nanocomposite synthesis, characterization and flammability properties. Mater. Chem. Phys..

[16] Becker C.M., Gabbardo A.D., Wypych F., Amico S.C. (2011). Mechanical and flame-retardant properties of epoxy/Mg-Al based LDH composites. Compos. Part A.

[17] Montero B., Bellas R., Ramirez C., Rico M., Bouza R. (2014). Flame retardancy and thermal stability of organic-inorganic hybrid resins based on polyhedral oligomeric silsesquioxanes and montmorillonite clay. Compos. Part B.

[18] Qina H., Zhanga S., Zhaoa C., Fenga M., Yanga M., Shub Z., Yang S. (2004). Thermal stability and flammability of pol-ypropylene/montmorillonite composites. Polym. Degrad. Stab..

[19] Rooj S., Das A., Thakur V., Mahaling R.N., Bhowmick A.K., Heinrich G. (2010). Preparation and properties of natural nanocomposites based on natural rubber and naturally occurring halloysite nanotubes. Mater. Design.

[20] Liu M., Jia Z., Jia D., Zhou C. (2014). Recent advance in research on halloysite nanotubes-polymer nanocomposites. Prog. Polym. Sci.

[21] Du M., Demin Jiab B.G. (2010). Newly emerging applications of halloysite nanotubes: A review. Polym. Int..

[22] Wang Z.Z., Zhou S., Hu Y. (2009). Intumescent flame retardation and silane crosslinking of PP/EPDM elastomer. Polym. Adv. Technol..

[23] Zhou S., Wang Z.Z., Gui Z., Hu Y. (2008). A study of the novel intumescent flame-retarded PP/EPDM copolymer blends. J. Appl. Polym. Sci..

[24] Lu H., Yanga W., Zhouga S., Xinga W., Songa L., Hua Y. (2010). Preparation and flammability of EPDM/PP/ Mg(OH)_2_ dynamic vulcanizates. Polym. Adv. Technol.

[25] Yu L., Wang W.J., Xiao W.D. (2004). The effect of decabromodiphenyl oxide and antimony trioxide on the flame retardation of ethylene−propylene−diene copolymer/polypropylene blends. Polym. Degrad. Stab..

[26] Chang Z.H., Guo F., Chen J.F., Yu J.H., Wang G.Q. (2007). Synergistic flame retardant effects of nano-kaolin and nano-HAO on LDPE/EPDM composites. Polym. Degrad. Stab..

[27] Jia S.J., Zhang Z.C., Du Z.W., Teng R.R., Wang Z.Z. (2003). A study of the dynamic flammability of radiation cross-linked flameretardant HDPE/EPDM/silicon-elastomer compound. Radiat. Phys. Chem..

[28] Beyer G. (2002). Nanocomposites: A new class of flame retardants for polymers. Plast. Addit. Compd..

[29] Pol V.G., Pol S.V., Gedanken A. (2005). Novel synthesis of high surface area silicon carbide by RAPET (reactions under autogenic pressure at elevated temperature) of organosilanes. Chem. Mater..

[30] Kumar S., Panda B.P., Mohanty S., Nayak S.K. (2020). Effect of silicon carbide on the mechanical and thermal properties of ethylene propylene diene monomer-based carbon fiber composite material for heat shield application. J. Appl Polym. Sci..

[31] Anancharoenwong E., Marthosa S., Suklueng M., Niyomwas S., Chaiprapat S. (2020). Effect of silicon carbide on the properties of natural rubber blends with EPDM rubber. Int. J. Integr. Eng..

[32] Gu J., Zhang Q., Dang J., Zhang J., Chen S. (2009). Preparation and mechanical properties researches of silane coupling reagent modified β-silicon carbide filled epoxy composites. Pol. Bull..

[33] Guo Z., Kim T.Y., Lei K., Pereira T., Sugar J.G., Hahn H.T. (2008). Strengthening and thermal stabilization of polyurethane nanocomposites with silicon carbide nanoparticles by a surface-initiated-polymerization approach. Compos. Sci. Technol..

[34] Todorova Z., Dishovsky N., Dimitrov R., El-Tantawy F., Abdel Aal N., Al-Hajry A., Bououdina M. (2008). Natural rubber filled SiC and B4C ceramic composites as a new NTC thermistors and piezoresistive sensor materials. Polym. Compos..

[35] Kueseng K., Jacob K.I. (2006). Natural rubber nanocomposites with SiC nanoparticles and carbon nanotubes. Eur. Polym. J..

[36] Alam M.N., Kumar V., Potiyaraj P., Lee D.J., Choi J. (2022). Synergistic activities of binary accelerators in presence of magnesium oxide as a cure activator in the vulcanization of natural rubber. J. Elastomers Plast..

[37] Laskowska A., Marzec A., Zaborski M., Boiteux G. (2014). Reinforcement of carboxylated acrylonitrile-butadiene rubber (XNBR) with graphene nanoplatelets with varying surface area. J. Polym. Eng..

[38] Masek A., Plota A. (2021). Influence of a natural plant antioxidant on the ageing process of ethylene-norbornene copolymer (Topas). Int. J. Mol. Sci..

[39] Szadkowski B., Marzec A., Rybiński P., Żukowski W., Zaborski M. (2020). Characterization of ethylene–propylene composites filled with perlite and vermiculite minerals: Mechanical, barrier, and flammability properties. Materials.

[40] Masłowski M., Miedzianowska J., Strzelec K. (2019). Hybrid straw/perlite reinforced natural rubber biocomposites. J. Bionic. Eng..

[41] Berahman R., Raiati M., Mazidi M.M., Paran S.M.R. (2016). Preparation and characterization of vulcanized silicone rubber/halloysite nanotube nanocomposites: Effect of matrix hardness and HNT content. Mater. Des..

[42] Szadkowski B., Marzec A., Zaborski M. (2020). Use of carbon black as a reinforcing nano-filler in conductivity-reversible elastomer composites. Polym. Test..

[43] Szadkowski B., Marzec A., Zaborski M. (2019). Effect of different carbon fillers on the properties of nitrile rubber composites. Compos. Interfaces.

[44] Namasivayam M., Shapter J. (2017). Factors affecting carbon nanotube fillers towards enhancement of thermal conductivity in polymer nanocomposites: A Review. J. Compos. Mater..

[45] Rybinski P., Syrek B., Marzec A., Szadkowski B., Kusmierek M. (2021). Effects of basalt and carbon fillers on fire hazard, thermal, and mechanical properties of EPDM rubber composites. Materials.

[46] Sowinska A., Maciejewska M., Guo L. (2020). Effect of SILPs on the vulcanization and properties of ethylene–propylene–diene elastomer. Polymers.

[47] Ismail H., Othman N., Komethi M. (2012). Curing characteristics and mechanical properties of rattanpowder-filled natural rubber composites as a function of filler loading and silane coupling agent. J. Appl. Polym. Sci..

[48] Shiva M., Dallakeh M.K., Ahmadi M., Lakhi M. (2021). Effects of silicon carbide as a heat conductive filler in butyl rubber for bladder tire curing applications. Mater. Today Commun..

[49] Alam M.N., Kumar V., Potiyaraj P., Lee D.-J., Choi J. (2021). Mutual dispersion of graphite–silica binary fillers and its effects on curing, mechanical, and aging properties of natural rubber composites. Polym. Bull..

[50] Kumar T.B., Haseebuddin M.R., Raghavendra N. (2018). Influence of SiC on mechanical, thermal, fire and wear studies of vinylester/glass fibre composites. Mater. Today.

[51] Wang Y., Wu J., Yin Y., Han T. (2020). Effect of micro and nano-size boron nitride and silicon carbide on thermal properties and partial discharge resistance of silicone elastomer composite. IEEE Trans. Dielectr. Electr. Insul..

[52] Tian H., Yao Y., Ma S., Wu J., Xiang A. (2017). Enhanced thermal stability and flame resistance of polyurethane-imide foams by adding silicon carbide. Adv. Polym. Technol..

[53] Du M., Guo B., Jia D. (2006). Thermal stability and flame retardant effects of halloysite nanotubes on poly (propylene). Eur. Polym. J..

[54] Kharazi A.P., Jalali-Arani A. (2017). Preparation of silicon carbide nanoparticle/butadiene rubber/silane nanocomposites; Structural, mechanical, tribological and thermal properties. J. Marcomol. Sci. Phys..

